# Patients’ confidence in treatment decisions for early stage non-small cell lung cancer (NSCLC)

**DOI:** 10.1186/s12955-020-01496-9

**Published:** 2020-07-18

**Authors:** Cecilia Pompili, Patricia Holch, Zoe Rogers, Kate Absolom, Beverly Clayton, Kevin Franks, Hilary Bekker, Galina Velikova

**Affiliations:** 1 Patient Centred Outcomes Research, Leeds Institute for Medical Research at St James’s, University of Leeds, Leeds, UK; 2 St James’ Institute of Oncology, Beckett Street, Leeds, LS9 7TF UK; 3 Department of Psychology, Leeds School of Social Sciences, Leeds Beckett University, Leeds, UK; 4 Leeds Teaching Hospital, Leeds, UK; 5 Leeds Unit of Complex Intervention Development (LUCID), School of Medicine, University of Leeds, Leeds, UK

**Keywords:** Shared decision making, Informed decision making, Self-efficacy, Lung cancer, Radiotherapy, Surgery

## Abstract

**Background:**

In early-stage Non-Small Cell Lung Cancer (NSCLC) patients, little is known about how to measure patient participation in Shared-Decision Making (SDM). We examined the psychometric properties and clinical acceptability of the Decision Self-Efficacy scale (DSE) in a cohort of patients undergoing to Stereotactic Ablative Radiotherapy (SABR) or Video-assisted Thoracoscopic Surgery (VATS) to capture patient involvement in treatment decisions.

**Methods:**

In the context of a prospective longitudinal study (Life after Lung Cancer-LiLAC) involving 244 patients with early-stage NSCLC, 158 (64.7%) patients completed the DSE either on paper or electronically online prior to treatment with SABR or VATS pulmonary resection. DSE psychometric properties were examined using: principal components analysis of item properties and internal structure, and internal construct validity; we also performed a sensitivity analysis according to Eastern Cooperative Oncology Group Performance Status (ECOG PS), gender, age and treatment received (VATS or SABR) difference.

**Results:**

Exploratory factor analysis using polychoric correlations substantiated that the 11 item DSE is one scale accounting for 81% of the variance. We calculated a value of 0.96 for Cronbach’s alpha for the total DSE score.

DSE scores did not differ by gender (*p* = 0.37), between the two treatment groups (*p* = 0.09) and between younger and older patients (*p* = 0.4). However, patients with an ECOG PS > 1 have a DSE mean of 73.8 (SD 26) compared to patients with a PS 0–1 who have a DSE mean of 85.8 (SD 20.3 *p* = 0.002).

**Conclusion:**

Findings provide preliminary evidence for the reliability and validity of the DSE questionnaire in this population. However, future studies are warranted to identify the most appropriate SDM tool for clinical practice in the lung cancer treatment field.

## Introduction

The most effective treatment options for curative lung cancer are video assisted thoracoscopic (VATS) resection or stereotactic ablative body radiotherapy (SABR) for patients unfit for surgery. The treatment effectiveness is evaluated on survival data [1, 2] and presently there is little information about the effects of these treatments on patients’ quality of life (QoL) [3, 4]. About 44% of patients diagnosed with lung cancer are aged 75 and over [5] and commonly have multiple comorbidities with 54% presenting with three or more [6] reducing their eligibility for surgery [6, 7]. This makes the patient’s decision to proceed with one of these treatments more complex, especially for those patients at higher medical risk.

Although current treatment guidelines do not recommend SABR as first-line treatment for Non-Small Cell Lung Cancer (NSCLC) moderate risk patients [1], multiple observational studies have suggested therapeutic equipoise exists between SABR and surgery in those patients with multiple comorbidities [8, 9]. The lack of long term QoL data from these two treatments has highlighted the importance of understanding whether a truly informed, shared treatment decision between patients and clinicians can be made. The quality of sharing this decision is highly dependent on the interaction in the patient-physician consultation, and the type of the information given.

It is unclear what needs patients have when faced with a choice between treatment options as often, improved knowledge about surgery does not necessarily translate into a more proactive attitude towards decision-making in the lung cancer setting [10]. It has been reported in lung cancer surgery, that many patients may wish to defer decisions about treatment to their medical team [10]. On the other hand, physicians have to be confident they have the skills to best support patients in a shared-decision making. In a Dutch survey, 30% of surgeons stated they are not properly trained to implement SDM in clinical practice [11]. Recent research in this field also confirmed that as result of the lack in standardized approach and deep understanding of SDM process, only 28.9% patients have been offered both treatment options for treatment of early-stage NSCLC [12].

Working towards patient and physician shared decision-making (SDM) for treatment of curative NSCLC in clinical practice will require the development of guidelines and their integration into existing decisional algorithms. The last two decades have witnessed an increasing number of trials investigating the overall lack of concordance between physician and patient perceptions of the decisional context in many clinical areas including lung cancer management. The majority of these trials have shown that concerns and treatment strategies were insufficiently discussed between the patients and physicians [12, 13].

Over the last 30 years, a number of measures have been developed to assess components of patient informed, values-based choices [14]. While, many different measures are available, the degree to which these measures are fully validated varies significantly. Currently, there is paucity of evidence assessing the views of patients with stage I NSCLC on aspects of shared decision-making (SDM).

To support proactively patients with cancer making decisions with physicians [14], a first step is to explore factors associated with patient confidence in involvement in the SDM process. Self-efficacy is a psychological construct referring to an individual’s judgment of the confidence to carry out a specific task in order to produce a desired outcome [12]. Furthermore, studies have shown self-efficacy has direct and indirect effect on QoL in patients with resected lung cancer [15].

Our aim was to assess the psychometric validity and sensitivity of the decision self-efficacy scale (DSE) in a cohort of early stage NSCLC patients undergoing SABR or VATS anatomical lung resection.

## Methods

### Design and sample

This design is a secondary analysis of data from a prospective longitudinal study of patients offered Stereotactic ablative radiotherapy (SABR) or Video-Assisted Thoracoscopic Surgery (VATS) for NSCLC in a large urban regional cancer centre (UK) between March 2017 to March 2018.

This study assesses the utility of a patient reported measure of decision making confidence carried out as part of the Life after Lung Cancer (LiLAC) study [4]. LiLAC used validated Patient-reported outcome measures (PROMs) to describe the trajectory of Quality of Life (QoL) following VATS or SABR treatment. This study received ethical approval from NRES Yorkshire and the Humber- Leeds East Research Ethics Committee (REC Ref: 16/YH/0407), and is registered in Clinicaltrial.gov database.

### Procedures

All participants completed (online or on paper) a set of questionnaires before their treatment (between 1 to 20 days before) to capture their preoperative QoL and Decision efficacy. Patients at this point have been already allocated to a treatment group as per multi-disciplinary team meeting (MDT) decision.

The Decision Self-Efficacy Scale (DSE) measures self-confidence or belief in one’s ability to make informed decisions and participate in shared decision making with health professionals [16]. It is a 11-item instrument with a five-point response scale ranging from 0 (not at all confident) to 4 (very confident). An example question is: *‘I understand the information enough to be able to make a choice’)*. The psychometric properties report a Cronbach alpha coefficient of 0.92, and the scale has been shown to be correlated with select subscales of the Decisional Conflict scale (DSC) [i.e., feeling informed (0.47) and supported (0.45) sub-scales] [17]. Scores are linearly transformed: score of 0 means ‘extremely low self-efficacy’ and a score of 100 means ‘extremely high self-efficacy’. Missing values are imputed using a single imputation method as recommended if the amount of missing data is below 10% [18]. Specifically, as DSE is a single scale, we follow the recommended approach used by most commonly used PROMs [19]: if at least half of the items from the scale have been answered, it is assumed that the missing items have values equal to the average of those items that are present for the respondent.

The following baseline demographic and clinical variables were collected: age, sex, forced expiratory volume in 1 s (FEV1) expressed in percentage of predicted value, Eastern Cooperative Oncology Group Performance Status (ECOG PS), diffusing capacity of the lung for carbon monoxide (DLCO) expressed in percentage of predicted value, treatment type and smoking habit. Performance status represents a holistic assessment of a patient’s functional capacity, which reflects the additive physical, physiological and psychological effects of the disease process. The two most commonly used measurement instruments to assess performance status or fitness for cancer treatments include the Eastern Cooperative Oncology Group Performance Status (ECOG PS) [20] and Karnofsky Performance Status (KPS) [21] scales. Although the two scales have been shown to be interchangeable, we have used the ECOG scale is for its simplicity and interobserver reproducibility [22, 23]. ECOG uses 5 points score to assess PS and is considered simple tool to use in daily clinical practice [20].

### Analysis

The study sample demographic and clinical characteristics were summarised using descriptive statistics.

An exploratory factor analysis (EFA) was performed to examine the underlying factors of the DSE. The minimum recommended sample size to conduct a EFA is 100 [24].

*An exploratory (principal axis) factor analysis* was conducted on the 11 DSE items from *N* = 158 cases items using IBM SPSS version 24. In addition to the total variance explained, the scree plot, eigenvalues and parallel analysis were assessed to verify the factor structure of DSE in this cohort. The factor analysis was performed using SPSS (IBM) Version 24 and for further exploration with parallel analysis and polychoric correlations ‘FACTOR’ Version 10.10.03 (Rovira i Virgili University, Spain). We used STATA 15.0 (Stata Corp., College Station, TX, USA) statistical software to analyse (1) descriptive statistics, (2) floor and ceiling effects (% scores at the minimum and maximum values), and (3) internal reliability (Cronbach’s alpha, corrected item-total correlations).

Although in the development studies Bunn [25] did not specify criteria for defining floor and ceiling effects, we employed the widely used N 15% of minimum/maximum scores cut-off [26]. Following convention, we considered Cronbach’s alpha’s ≥ 0.70 to indicate acceptable internal consistency [27]. Feasibility was also assessed, in terms of proportion of missing data (“prefer not to answer” responses for the dataset overall and per item). We considered that < 5% missing data overall was acceptable, although we acknowledge there is no established consensus on this issue, with recommended criterion values ranging from 5 to 20% [28].

For highly related constructs, moderate to strong associations (r ~ +/− 0.40 to 0.80) between these determinants and the factors of the DSE were expected [29].

Sensitivity of the module was assessed by means of known group differences according to the Performance Status, gender, age and treatment received (VATS or SABR).

As all the DSE variables were not normally distributed, they were compared across groups by the Mann–Whitney *U* test.

## Results

### Participant characteristics

A total of 244 patients consented to the study of which 158 (64.7%) returned the Decision Self-Efficacy Scale at baseline. Of these, 73 patients were treated using SABR, and 85 had VATS. We did not find any baseline difference between patients who completed the DSE (*n* = 158) and those who didn’t (*N* = 86) in terms of age (p:0.17), gender (*p* = 0.34) and PS > 1(*p* = 0.23). A table including characteristics of the whole cohort of patients included into the Lilac study are provided in Supplementary File 1. Patients treated with SABR were older, with more comorbidities, lower FEV1 and DLCO values and higher PS. These differences were, however expected as the SABR treatment was indicated to those patients who were not physiologically fit for surgery.

The baseline clinical characteristics of the participants included in this study are in listed in Table 1.

**Table 1:** Baseline demographic and clinical characteristics of participants *N* = 158

Variable	All patients with DSE (*n* = 158)	SABR (*n* = 73)	Surgery (*n* = 85)
Treatment (surgery, %)	85 (53.8)		
Gender (male, %)	69 (43.6)	26 (35.6)	43 (50.5)
Age (years, SD)	72.4 (8.6)	74.5 (9.3)	70.5 (7.5)
Comorbidity (yes, %)	135 (85.4)	67 (91.7)	68 (80)
FEV1% (SD)	83.5 (25.1)	75.5 (27.4)	89.2 (21.8)
DLCO% (SD)	77.6 (22.2)	69.6 (22.8)	83.5 (19.9)
Currently smoking (n, %)	34 (22.6%)	19 (27.1)	15 (18.7)
PS > 1 (n, %)	104 (62.4)	39 (53.4)	15 (17.6)
DSE (Mean, SD)	81.7 (23)	79.5 (23)	83.6 (22.9)

Results are expressed as mean and standard deviation (SD) for numeric variables and as count and percentages for categorical variables. FEV1: forced expiratory volume in 1 second; diffusing capacity of the lung for carbon monoxide (DLCO) expressed in percentage of predicted value, Eastern Cooperative Oncology Group Performance Status (PS).

Mean value of the DSE was 81.7 (SD 23). In the Surgical group the mean score was 83.6 (SD 22.9) and in the SABR group was 79.5 (SD 23). DSE is the main score representing the overall efficacy in making the decision.

### Exploratory factor analysis

#### First stage

An EFA analysis initially using Pearson’s correlations was conducted on the 11 items using principal axis factoring and an oblique (direct oblimin) as it was expected that the factors would not be independent. The Kaiser-Meyer-Olkin (KMO) measure verified sampling adequacy for the analysis (KMO = .91) well above the minimum criterion of .50, in addition all KMO values for individual items were ≥ .88 and Bartlett’s test of sphericity was also significant at *p* < .001. An initial analysis was run and factors retained using three conventions, i) Kaiser’s criterion of eigenvalues over 1, ii) inflections in the scree plot and ii) parallel analysis [30]. Two factors had Eigen values over Kaisers criterion of 1 (7.91 and 1.02 respectively) and the scree plot depicted two inflections confirming Eigen values over 1. However, to further explore whether second factor (just over Kaisers threshold > 1) was not due to chance, a parallel analysis was performed using a permutation of the raw data [31]. This analysis strongly suggested rejection of the second factor with 95 percentiles of random % of variance (18.28) generated which is greater than the real data % of variance (6.78).

#### Secondary

To further explore the DSE we used principal axis factoring with polychoric correlations and the Kaiser-Meyer-Olkin (KMO) was again adequate (KMO = .60) and Bartlett’s test of sphericity was also significant at *p* < .001. This substantiated that the DSE is a one factor scale with one Eigen value identified over the threshold explaining 81% of the variance (Table 2), therefore, a factor loading plot was not computed.

**Table 2:** Extracted eigen values and % of variance after polychoric correlations (*N* = 158)

Eigen value	Total	% of Variance	Cumulative %
**1**	**9.00**	**81.00**	**81.00**
2	0.74	7.00	88.00
3	0.40	3.00	
4	0.24	2. 00	
5	0.17	1.00	
6	0.15	1.00	
7	0.12	0.01	
8	0.10	0.01	
9	0.05	0.01	
10	0.02	0.01	
11	0.01	0.01	

#### Construct validity

The decision self-efficacy scale performed well in terms of psychometrics in this sample (Table 3): we calculated a value of 0.96 for Cronbach’s alpha for the total DSE score. The overall amount of missing data was 1.5% of the dataset which is in line with the developer’s data [25]. However, a notable ceiling effects with a significant proportion scoring substantially over the whole DSE (Table 3).

**Table 3:** Descriptive statistics, floor and ceiling effects and internal reliability for the DSE

	N	Mean	(SD)	Floor effect (%min score)	Ceiling effect (%max score)
DSE	158	81.7	23	0.63%	32.9%

The items correlated significantly at *p* = 0.001. A determinant value of 2.09E-006 (above the necessary value of 0.00001) revealed that the level of collinearity would not be detrimental to the analysis therefore, no items were removed. The polychoric correlation matrix is provided in the Supplementary File 2.

### Known-group differences

Group comparisons revealed no significant mean differences between the two treatment groups in terms of overall self-efficacy score (DSE): SABR 79.5, Surgery 83.6 (*p* = 0.09). There were no statistical differences between the two groups for each of the eleven items either (Table 4).

**Table 4:** Total DSE and DSE individual questions scores in SABR and Surgical groups

Item	Surgery (*n* = 85) Mean (SD)	SABR (*n* = 73) Mean (SD)	*p* value
DSE total score	83.6 (22.9)	79.5 (23.1)	0.09
1. Get the facts about medication choices available to me	85.2 (23.5)	80.4 (26.1)	0.13
2. Get the facts about the benefits of each choice	83.5 (26.0)	79.1 (26.0)	0.10
3. Get the facts about the benefits and risks of each choice	84.1 (25.8)	80.1 (27.3)	0.21
4. Understand the information enough to be able to make a choice	84.4 (26.1)	82.8 (24.6)	0.33
5. Ask questions without feeling dumb	84.4 (30.8)	79.7 (30.8)	0.34
6. Express my concerns about each choice	83.5 (26.8)	78.0 (30.0)	0.16
7. Ask for advice	87.0 (26.8)	82.8 (26.3)	0.11
8. Figure out the choice that best suits me	84.1 (23.7)	79.1 (28.8)	0.35
9. Handle unwanted pressure from others in making my choice	80.8 (29.2)	77.3 (30.6)	0.51
10. Let the clinic team know what’s best for me	83.8 (22.0)	77.7 (31.0)	0.50
11. Delay my decision if I feel I need more time	79.1 (31.5)	76.7 (31.5)	0.88

Patients with the ECOG PS > 1, less fit, reported lower self-efficacy in making their decision during the preoperative period. Indeed, patients with PS > 1 have a DSE mean of 73.8 (SD 26) compared to patients with a PS 0–1 who have a DSE mean of 85.8 (SD 20.3 *p* = 0.0024).

No statistically significant differences between DSE scores for men and women were evident (*p* = 0.37). Male patients had a mean DSE value of 84.0 (SD 21.3) and female of 79.9 (SD 24.2).

Similarly, when comparing DSE among younger and older people (using the cut-off above and below the median value of 72 years) no statistically significant differences were found (*p* = 0.4). In particular, older people > 72 years had a DSE mean of 82.5 (SD 23.7) and younger people < 72 years 81 (SD 22.6).

## Discussion

We aimed to explore the psychometric properties of the DSE in a cohort of patients undergoing VATS resection or SABR therapy for NSCLC. Our findings suggest the DSE is valid: the 11-item measure has good internal consistency (α of 0.96), and is one scale explaining 81% of the variance. The developers’ recommendation for one scale is confirmed by the observed high internal consistency in our study, however, we would recommend further exploration of the DSE in other cancer populations.

The observed significant ceiling effect (over 15% of responses) should be noted. No data about ceiling or floor effect have been published for the DSE. However, a recent systematic review of existing measures of self-efficacy in cancer patients, reported that these psychometric properties were not often assessed [32]. The timing of assessment in our population (after the decision) may have affected the results.

Almost 70% of the sample completed the decision self-efficacy scale demonstrating that the collection of this data is possible in this population. Furthermore, the overall proportion of missing data was low (1.5%), indicating that DSE was acceptable to patients.

There was no difference in efficacy with decision making by treatment type in this study. Patients with poor clinical performance status were more likely to be less confident in making their decision for treatment. We know patients who have a worse PS and limited functional capacity tend to have more difficulty tolerating rigorous NSCLC cancer treatments, i.e. they have less favourable outcomes than fitter patients regardless of treatment type [33, 34]. One explanation can be that regardless of the treatment type when patients are less independent physically (as in those with higher PS score), they have more conflict or difficulty deciding about the best treatment to meet their needs. In addition, performance status and NSCLC cancer stage were significantly more influential than biological age when recommending treatments [35]. In this sense, physicians may tend to involve patients with a higher PS score less in the decision-making process, presumably with concerns about higher expected morbidity and mortality. In those cases, patients may perceive similarly less confidence in making that decision which is more “physician-driven”. Another possible explanation can be related to the fact that patients more compromised were never involved in discussions about possible treatment alternative i.e. surgery: this may have influenced their efficacy in making the treatment decision as their opinion may have not played a role at all in all the course of the decision-making process.

The decision efficacy scale has previously been evaluated in patient populations referring to patient’s making decision regarding immunizations, screening, hormone replacement therapy, blood pression medications adherence [36–38] suggesting a good understanding and applicability of this questionnaire in field where difficult decisions need to be taken [17]. For NSCLC patients it would be useful to investigate if they, not only have the self-efficacy but the ability to ask questions and clearly express their values and prediction of outcomes.

O’Connor developed within the same conceptual framework of the DSE a 16-item Decisional Conflict Scale rated on a Likert scale measuring the uncertainty in choosing options, modifiable factors contributing to uncertainty (information, values and social support) [17].

In situations where the evidence available is not clearly defined and the long-term benefits are still undetermined (as with SABR Vs VATS), the understanding of conflict in difficult decisions may be more relevant to help identifying patient’s needs and possibly develop tailored decision aids.

The implementation of a decision aid in the field of early stage NSCLC has the potential also to streamline the pre-treatment pathway and reducing the referral to a second speciality opinion in these patients care. The decision conflict scale could be clinically more applicable to the conflictual choice between surgery and SABR especially for those borderline patients, where there is a clear equipoise in terms of risks and benefit, an observation highlighted in the SABRTooth trial [39]. In high risk patients where the surgery has not been completely excluded by objective parameters, the decision should be tailored and supported by the medical team but also with the use of validated decision aids, as successfully demonstrated in other specialities [40]. It would also be important to investigate and measure the involvement in decision and the ability to access and understand information [41]. However, we must be aware that a good decision often doesn’t correspond to a good outcome: indicators of good decision making may include reduced uncertainty, improved knowledge, more realistic expectations, improved clarity of values and value congruence with the decision; reduced decision delay; improved adherence to the decision, and efficacy [17, 42]. Understanding the latter, can have crucial clinical implications in the development of a decision aid and ultimately help people considering their options and making the best decision for themselves.

### Limitations

The return rate of a 67% is reasonable for self-report questionnaires, but there may be a possibility those who did not complete the questionnaire had different experiences which could impact on the findings. Further investigation of its psychometric properties in samples which include a wider group of patients is advised, and methods to enable further validation data.

The study had a relatively small sample size, and was performed in a single centre. Certainly, it seems that the processes to choose between treatment types in NSCLC cancer are similar, suggesting the same type of information about the risks and benefits and long-term consequences of both treatments should be presented equally by clinicians to support informed choice. Alternatively, it may be that this questionnaire is not as sensitive to the differences between the different types of choices as other measures, e.g. decisional conflict scale. Certainly, patients found it difficult to make a treatment choice, regardless of treatment type, suggesting a decision aid might be helpful for patients to reach a decision with greater confidence. However, the questionnaire has not up till now been tested in a cancer population. Previously the DSE had been utilised with menopausal woman and psychiatric patients [16, 19], thus limiting the comparability of our findings.

Another limiting factor of our study design is that we collected the questionnaires after patients made their final decision; it may be that there are more patients who do not feel involved in their decision making earlier in the treatment pathways. This data collection method may, in part, explain the high ceiling effects as people’s views and exposure to further information will change from diagnosis to after treatment.

## Conclusions

We demonstrated that the collection of decision efficacy questionnaire in a population of NSCLC cancer patients facing difficult decision is feasible. We provided evidence for the validity of the DSE as a11-item measure comprising one scale. Our results demonstrated that the DSE questionnaire discriminates between the low and high PS of patients. This confirms the importance to identify those patient subgroups which will benefit from programmes aimed to improve their participation in treatment decision-making contexts.

The conflict, more than the social and emotional component, of the difficult-decision making may be more relevant when evaluating the routine data collection in complex clinical area like this one [43]. This may help identify people with greater need of help and thus enable specific support in making decisions and will help in the future tailoring of specific decision aids.

Our study findings may also inform future investigations around decision making within complex care and the resources required to reliably collect information on SDM process in clinical practice.

## Supplementary information

**Additional file 1: Supplementary File 1.** Table describing the characteristics of the whole study sample and the patients who completed the DSE questionnaire.

**Additional file 2: Supplementary File 2.** Polychoric correlational matrix related to exploratory factor analysis.

## Data Availability

The data that support the findings of this study are available from Cecilia Pompili but restrictions apply to the availability of these data, which were used under license for the current study, and so are not publicly available. Data are however available from the authors upon reasonable request and with permission of Cecilia Pompili.

## Abbreviations

NSCLC: Non-Small Cell Lung Cancer
SDM: Shared-Decision Making
DSE: Decision Self-Efficacy scale
LILAC: Life after Lung Cancer
SABR: Stereotactic Ablative Radiotherapy
VATS: Video-assisted Thoracoscopic Surgery
PS: Eastern Cooperative Oncology Group Performance Status
MDT: Multi-disciplinary team meeting
PCA: Principal Component factor analysis
FEV1: Forced expiratory volume in one second
DLCO: Diffusing capacity of the lung for carbon monoxide
