# Supplementary material for: Patients’ confidence in treatment decisions for early stage non-small cell lung cancer (NSCLC)
Source: Health Qual Life Outcomes. 2020 Jul 18;18:237. doi: 10.1186/s12955-020-01496-9 (PMC7368734; doi:10.1186/s12955-020-01496-9)
Supplement: Supplementary file 1 — Additional file 1: Supplementary File 1. Table describing the characteristics of the whole study sample and the patients who completed the DSE questionnaire. [file 12955_2020_1496_MOESM1_ESM.docx]

Supplementary File 1: Table describing the characteristics of the whole study sample and the patients who completed the DSE questionnaire.

| Variable | SABR with DSE (n=73) | Total SABR included in the study (n=95) | Surgery with DSE (n=85) | Total Surgery included in the study (n=130) |
| --- | --- | --- | --- | --- |
| Gender (male, %) | 26 (35.6) | 37 (39) | 43 (50.5) | 62 (48) |
| Age (years, SD) | 74.5 (9.3) | 74.3 (9.2) | 70.5 (7.5) | 70.0 (8.8) |
| Comorbidity (yes, %) | 67 (91.7) | 89 (93.6) | 68 (80.0) | 99 (76.1) |
| FEV1% (SD) | 75.5 (27.4) | 76.6 (26.3) | 89.2 (21.8) | 87.9 (22.3) |
| DLCO% (SD) | 69.6 (22.8) | 70.9 (22.1) | 83.5 (19.9) | 83.4 (21.0) |
| Currently smoking (n, %) | 19 (27.1) | 25 (27.8) | 15 (18.7) | 30 (24.1) |
| PS >1 (n, %) | 39 (53.4) | 54 (56.8) | 15 (17.6) | 21 (16.1) |
