# Supplementary material for: Patients’ confidence in treatment decisions for early stage non-small cell lung cancer (NSCLC)
Source: Health Qual Life Outcomes. 2020 Jul 18;18:237. doi: 10.1186/s12955-020-01496-9 (PMC7368734; doi:10.1186/s12955-020-01496-9)
Supplement: Supplementary file 2 — Additional file 2: Supplementary File 2. Polychoric correlational matrix related to exploratory factor analysis. [file 12955_2020_1496_MOESM2_ESM.docx]

Supplementary File 2: Polychoric correlational matrix related to exploratory factor analysis

| correlation | Q1 | Q2 | Q3 | Q4 | Q5 | Q6 | Q7 | Q8 | Q9 | Q10 | Q11 |
| --- | --- | --- | --- | --- | --- | --- | --- | --- | --- | --- | --- |
| Q1 | 1.000 |  |  |  |  |  |  |  |  |  |  |
| Q2 | 0.991 | 1.000 |  |  |  |  |  |  |  |  |  |
| Q3 | 0.945 | 0.944 | 1.000 |  |  |  |  |  |  |  |  |
| Q4 | 0.834 | 0.853 | 0.894 | 1.000 |  |  |  |  |  |  |  |
| Q5 | 0.703 | 0.722 | 0.708 | 0.787 | 1.000 |  |  |  |  |  |  |
| Q6 | 0.730 | 0.745 | 0.791 | 0.834 | 0.929 | 1.000 |  |  |  |  |  |
| Q7 | 0.741 | 0.752 | 0.750 | 0.781 | 0.876 | 0.881 | 1.000 |  |  |  |  |
| Q8 | 0.773 | 0.777 | 0.805 | 0.815 | 0.818 | 0.838 | 0.802 | 1.000 |  |  |  |
| Q9 | 0.700 | 0.730 | 0.774 | 0.794 | 0.834 | 0.898 | 0.830 | 0.845 | 1.000 |  |  |
| Q10 | 0.716 | 0.731 | 0.761 | 0.716 | 0.755 | 0.797 | 0.745 | 0.886 | 0.775 | 1.000 |  |
| Q11 | 0.743 | 0.725 | 0.705 | 0.733 | 0.756 | 0.795 | 0.790 | 0.799 | 0.776 | 0.845 | 1.000 |
